# State Association of Health Officers of Texas

**Published:** 1901-04

**Authors:** 


I. J. Jones, M. D.

---

### Society Notes.

### STATE ASSOCIATION OF HEALTH OFFICERS OF TEXAS.

### Stenographic Report for the Texas Medical Journal, Official Organ of the Association.

The State Association of Health Officers of Texas met in the Knights of Pythias Hall, Austin, pursuant to the adjournment at Temple, on March 21, 1901, at 10 a. m. The temporary president of the Association, Dr. Thomas Moody, of Paris, not being present, the Association was called to order by Dr. I. J. Jones, of Austin. Dr. J. B. Massie, of Houston, was elected President *pro tem.* A committee of three was appointed, consisting of Drs. Jones of Austin, Campbell of Dallas, and Blythe of Mt. Pleasant, to draft a scheme of organization. The committee reported as follows:

1. The title of the organization shall be "The Health Officers Association of the State of Texas."

2. All State, county and city health officers, the mayors of incorporated cities and towns, county judges and sanitary officers, are eligible to membership.

3. The Association shall meet twice annually. Once at Austin on first Tuesday in February, and once at place selected by the Association, on the first Tuesday in August.

4. The officers of the Association shall be president, vice-president, and secretary-treasurer.

5. The secretary shall receive an annual salary of one hundred dollars.

6. The qualification for membership shall be a certificate of the official character of the applicant.

7. The dues shall be two dollars per annum, payable semi-annually.

JONES,
ANDREWS,
BLYTHE,
Committee.

The first business was the election of permanent officers. Dr. Massie was placed in nomination for permanent president, and on motion of Dr. Jones, the secretary was instructed to cast the ballot of the Association for Dr. Massie. Dr. Jones was nominated for vice-president, and on motion the secretary was instructed to cast the ballot of the Association for Dr. Jones. Dr. McCutchan, of Temple, was nominated for secretary, and on motion the president was instructed to cast the ballot of the Association for Dr. McCutchan.

A paper was read by Dr. Myers, of Seguin. Subject: "A Brief History of the Recent Outbreak of Smallpox in Guadalupe County, and Other Remarks."

Dr. Daniel, editor of the TEXAS MEDICAL JOURNAL, was asked by the chair to open the discussion. (Paper and discussion will appear next month.—ED.)

Dr. Jones moved that a committee be appointed for drawing up a memorial to the Legislature to pass a law to control the present epidemic of smallpox. the motion being duly seconded, was carried. Drs. Stallcup of Marion county, Campbell of Dallas, and Blythe of Titus, were duly appointed on that committee.

The meeting then adjourned till 2 p. m.

Meeting called to order at 2 o'clock by the president.

Dr. W. H. Blythe, of Titus, read a paper on the subject of "Cuban Itch." (Paper and discussion will appear in next issue. —Ed.)

Dr. Jones: "Yesterday I met a gentleman whom I consider it an honor to know; whose name is familiar to all—Dr. T. C. Osborne, of Cleburne. He was the first to write a clinical history of malarial hæmaturia. He was the first to suggest, in April, 1894, and publish in June, 1894, that bichloride of mercury is the best treatment for smallpox, and will prevent it the same as vaccination will; he also claims that smallpox is only a local disease of the skin. He requested me to ask all gentlemen present who had patients that would not submit to vaccination to use the bichloride of mercury as a prophylactic, and report progress at our next meeting. I, therefore, make this motion, that each and every health officer here, where vaccination of exposed persons cannot be obtained, will use bichloride of mercury and report progress at our next meeting.

Dr. Myers was of the opinion that health officers who have to come in contact with cases of smallpox should sponge their clothing with bichloride.

Dr. Daniel called attention to an article published in the Texas Medical Journal some time ago, by Dr. R. H. L. Bibb, Chief Surgeon Mexican National Railroad at Saltillo, Mexico, who had one hundred cases of smallpox whom he bathed thoroughly with bichloride. There were only two deaths. Dr. Bibb stated that the application arrested the disease immediately; that the papules turned black and fell off. Dr. Daniel thought the remedy well worth trying.

Dr. Graves (Travis county) differed with Dr. Bibb. He said that in a few cases bichloride decreases the fever and probably makes the pustules dry up quicker; yet he thought Dr. Bibb claimed too much for the baths.

Dr. Massie declared that he owed a debt of gratitude to Dr. Osborne, yet he thought the doctor too enthusiastic and extravagant in his claims for bichloride of mercury baths. "But," he says, "I

have used the remedy in all stages of the disease, and find that it modifies the disease; the pustlating is not so great, and the fever is reduced, and so the doctor is entitled to everlasting gratitude."

Dr. Jones gave a brief statistical report of the use of bichloride of mercury. He stated that in this State the use of bichloride has been universal; that during the past twelve months there had been 7,900 cases of smallpox, with a mortality of 1.27. In Louisiana the average death rate is 11 per cent. In the city of New Orleans, where they do not use the bichloride, it is 32.44, and in the city of Shreveport, where they use the bichloride, the death rate is 10 per cent. "So," he says, "I think we have very strong claims for the use of bichloride."

Dr. Hill (Trinity county) advocated the use of bichloride with ichthyol. He claimed that this treatment gives more comfort to the patient than any other treatment. He also maintained that where there is no secondary scab, there is no pitting. Defining a "secondary scab," he said it is a scale which forms after the scab has fallen off.

Dr. Graves said he had never seen a "secondary scab" in his treatment of smallpox.

Dr. Myers said he had seen a number of secondary scabs, but that they were not infectious.

Dr. Massie inquired of Dr. Hill (who had smallpox while at Tulane Medical College, New Orleans) the custom at the Tulane College in regard to opening pustules, and was told (by Dr. Hill) that the pustules were not opened. Dr. Graves agreed to this mode of treatment. Dr. Massie differed, maintaining that the opening of the pustules prevented pitting.

The president asked if there was a second to Dr. Jones' motion, and receiving same, the motion was carried.

Dr. Brownlee, of Burnet, read a very interesting paper on the subject of "General Health."

Dr. Massie said, in substance, that while we have smallpox spread over our country to an alarming extent, we must not allow it to engross our entire attention; that we have typhoid fever in our midst, as well as many other dangerous diseases, and that our people should be educated in the laws of general health, and that the physicians are the ones to do it.

Dr. Jones considered the paper very suggestive, because there has been no recognition of general sanitation, and the outgrowth of this medical association should be plans for general sanitation of the State. He called attention to the mosquito as a distributor of malaria, and the amount of good that petroleum properly applied to stagnant pools of water would do. He was interrupted by Dr. Hamilton, of Corsicana, who said that it was a mistaken belief that petroleum prevented mosquitoes; that in the city of Corsicana, oil floated on stagnant pools, and that mosquitoes were as bad there as anywhere.

Dr. Daniel tendered the pages of the TEXAS MEDICAL JOURNAL for the publication of the Association's papers and proceedings. Dr. Jones moved a vote of thanks to Dr. Daniel for his kind offer in the use of the JOURNAL, and on motion the TEXAS MEDICAL JOURNAL was made the official organ of the Association; and on motion of Dr. E. M. Thomas, of Williamson county, Dr. Daniel was made an honorary member of the Association.

Dr. Guinn, of Rusk, read a paper on "Compulsory Vaccination."

Dr. I. P. Sessions (Milam county) stated that they had had two hundred cases of smallpox in Rockdale. He did everything that could be done; put on a vigorous quarantine, and vaccinated everyone, and finally controlled the disease. He was of the opinion that the prevention of a further spread of the disease was due to the fact that he had everyone vaccinated. He said that the expense of quarantine had been a very serious question in his city, the question being as to who should pay for quarantine for incorporated towns.

Dr. Myers said that a law might be passed prohibiting any one who has not been vaccinated from going to the polls to vote; or that a law might be passed prohibiting marriage, unless the contracting parties have both been vaccinated, but that we could not get a law passed for *compulsory vaccination*.

Dr. Andrews, of Wharton, read a paper on "Strict Quarantine as it Appears on the Statutes." (The papers read at this meeting will appear in the JOURNAL later.—ED.)

Dr. McCutchan emphasized the importance of the quarantine of the State being focused in order to be systematized; for unless it is systematized, we can accomplish but little.

Dr. Massie considered the law as it appears on the statute book inadequate to control infectious diseases.

The committee appointed to prepare the memorial reported their memorial in readiness.

The memorial was read, and on motion of Dr. Myers it was adopted.

Dr. Massie said, "If we had the time, I would not be in favor of making any compromise in regard to compulsory vaccination; but we have not the time; we must try to secure one of the bills now pending."

Dr. Jones moved that every member of the Association communicate with his representative, and that the secretary send a copy of these resolutions to every county and city health officer who is not present.

Dr. Erwin (of Hearne, Texas) added to the motion that each member of the Association see his representative *before leaving Austin*. Carried.

Dr. Tabor (of Bryan, Texas) made a motion that a committee of three be appointed to convey the memorial to the House committee. The motion being carried, the president appointed on that committee Drs. G. R. Tabor of Brazos county, Campbell of Dallas county, and J. M. Andrews of Wharton county.

Dr. Jones moved that the president prepare an address, which the secretary is to furnish to every physician not present urging him to meet with us.

Dr. Grube of New Braunfels (Comal county) suggested that the address should set forth the purpose of the Association. Carried.

On motion a committee of three was appointed to draw up a memorial, or petition, to the Legislature, praying for more perfect quarantine laws.

The committee made the following report, which was adopted:

We, your committee appointed for the purpose of drafting a suitable memorial to the Legislature on the subject of quarantine legislation, beg leave to report the following memorial:

*To the Honorable Senate and House of Representatives of the State of Texas:*

We, your petitioners, The Health Officers Association of the State of Texas, beg leave to represent that there prevails in our State an extensive infection of smallpox. That this disease, while mild in character, heretofore, has caused the death of not less than seven hundred and fifty citizens of this State in the past two years. That in the neighboring States a far greater mortality has occurred. That this disease is likely to assume a more malignant form at any time.

That the present law of local quarantine is totally inadequate for the control of the disease for two reasons:

1. There is no method by which uniform action by all counties and cities of the State can be obtained. And that while one county or city is stamping out the disease, another is inactive, and incubating the infection.

2. No adequate method is provided for defraying the necessary expenses.

Now, therefore, we, your petitioners, would respectfully urge that your honorable bodies shall, before the end of the present session, pass the pending quarantine bills with such amendments as are necessary to carry out the suggestions of this memorial.

T. H. Stallcup, Marion Co.,
W. H. Blythe, Titus Co.,
Peyton L. Campbell, Dallas Co.,
Committee.

The Association then adjourned, to meet in Dallas the first Tuesday in August.

